# Comparison of continuous glucose monitoring profiles during the day and night in healthy and diabetic cats

**DOI:** 10.1177/1098612X251376606

**Published:** 2025-11-08

**Authors:** Carole Schuppisser, Filippo Ferri, Claudia E Reusch, Tony Glaus, Eric Zini

**Affiliations:** 1 Clinic for Small Animal Internal Medicine, Vetsuisse Faculty, University of Zürich, Switzerland; 2 AniCura Istituto Veterinario Novara, Granozzo con Monticello, Italy; 3 Studio Veterinario Associato Vet2Vet di Ferri e Porporato, Orbassano, Italy; 4 Department of Animal Medicine, Production and Health, University of Padova, Legnaro, Italy

**Keywords:** Nocturnal, diurnal, hypoglycaemia, glucose curves, diabetes mellitus management

## Abstract

**Objectives:**

This study aimed to detect hypoglycaemia episodes and evaluate glucose dynamics and glycaemic variability in insulin-treated diabetic and healthy cats using a continuous glucose monitoring system (CGMS). Blood glucose curves are useful for managing insulin-dependent diabetes mellitus (DM) but may miss the nadir and peak.

**Methods:**

A CGMS with a 7-day recording period (range 2.2–22.2 mmol/l) was implanted in six healthy and 10 insulin-treated diabetic cats to obtain 24-h glucose curves. For each cat, mean, minimum and maximum glucose concentration, SD and coefficient of variation (CV) were calculated for the daytime and night-time.

**Results:**

Most diabetic cats removed sensors prematurely, but 24-h curves were obtained in all; the only adverse effect of the device was mild local skin irritation. In healthy cats, nights had higher SD (0.6 mmol/l [range 0.2–0.8] vs 0.4 mmol/l [range 0.2–0.6]; *P* = 0.037) and CV (13% [range 5–23] vs 10% [range 4–16]; *P* = 0.041) compared with the days; mean, minimum and maximum glucose concentration showed no diurnal-nocturnal differences. In diabetic cats, no differences were observed for mean, minimum and maximum glucose concentrations, SD and CV. Hypoglycaemia episodes (<3.5 mmol/l) occurred in five healthy and four diabetic cats, either during the day or night. Compared with well-controlled diabetic cats, those with moderate to poor control had higher mean and maximum glucose concentrations during the 24 h and had higher SD during the day than at night.

**Conclusions and relevance:**

Continuous glucose monitoring revealed increased nocturnal glycaemic variability in healthy cats but not in diabetic cats. Furthermore, cats with moderately to poorly controlled DM had higher diurnal glycaemic variability than those well controlled. Low glucose concentrations occurred in both groups and at any time, emphasising the benefit of 24-h glucose curves in diabetic cats.

## Introduction

Blood glucose curves are useful for managing insulin-dependent diabetes mellitus (DM) in humans,^1–4^ dogs^5^ and cats,^6–8^ particularly in patients with poorly regulated DM. Continuous glucose monitoring systems (CGMS) have become valuable tools in veterinary medicine, offering real-time, non-stop interstitial glucose measurements compared with traditional daytime intermittent blood draws.^9–12^ However, the latter is still commonly used in daily clinical practice, even if it may miss glucose nadirs and peaks. In diabetic humans, up to 55% of all severe hypoglycaemia occurs at night.^13,14^ The incidence of nocturnal hypoglycaemia in diabetic dogs was recently published, and no differences were found between night-time and daytime glucose parameters.^15^ In cats, nocturnal glucose profiles at home have not been studied so far; understanding the frequency and patterns of nocturnal hypoglycaemia is critical for improving DM management, as hypoglycaemic episodes at night may go unnoticed and lead to adverse outcomes.

Glycaemic variability, characterised by fluctuations in blood glucose concentrations within and between days, including episodes of hypo- and hyperglycaemia, is a recognised risk factor for DM-related complications in humans.^16–22^ Commonly used indicators for glycaemic variability are SD and coefficient of variation (CV).^19,23^ The SD describes the dispersion of data on both sides of the mean and is considered the simplest approach for evaluating glucose variability in human diabetics.^19,23,24^ The CV, which is the ratio of the SD to the mean, provides a standardised measure of variability. This parameter allows for the comparison of glucose variability between patients with different mean blood glucose concentrations. Using the parameter SD, glycaemic control is considered acceptable when in type 1 DM the SD is less than the mean blood glucose/2, whereas in type 2 DM, the SD should be less than mean blood glucose/3.^19,20,24^ Although recent publications have begun addressing glycaemic variability in cats, there is still limited comprehensive understanding of the topic. In particular, knowledge about glycaemic variability in the daytime compared with night-time in cats is scant. Our group recently showed that glycaemic variability is increased in diabetic cats with insulin-induced post-hypoglycaemic hyperglycaemia, which is associated with higher insulin dose, higher fructosamine concentrations and a lower remission rate.^25^

Interstitial glucose concentrations measured by CGMS closely correlate with plasma glucose concentrations.^26,27^ The CGMS iPro2 (Medtronic) is designed to measure and record interstitial glucose values in humans for up to 7 days. This device may be suitable for use in diabetic cats in their home environment because it does not involve a monitor. In our previous study, the iPro2 was shown to be reliable in cats in the normo- and hypoglycaemic range.^28^

With this background, the aim of this study was to evaluate the continuous glucose profiles and glycaemic variability during both daytime and night-time in diabetic and healthy cats at home, utilising the CGMS iPro2.

## Materials and methods

### Healthy cats

Six healthy purpose-bred domestic shorthair cats were used as control cats. The study was approved by the State Veterinary Office of Zürich (application number 110/2014, 6 June 2014).

### Insulin-treated diabetic cats

Client-owned insulin-treated diabetic cats were prospectively recruited to participate in this study if concurrent diseases were ruled out based on unremarkable physical examination and laboratory analyses (including haematology, biochemical profile, urinalysis, serum total thyroxine and insulin-like growth factor 1).

### Continuous glucose measurement and data analysis

The CGMS iPro2 system comprises a disposable sensor, a recorder and a docking station. The sensor measures the glucose concentration in the interstitial fluid via an enzymatic reaction that generates a small electrical current. This signal is subsequently converted to a glucose concentration (mmol/l) and saved to the recorder. The iPro2 measures glucose concentration in the range of 2.2–22.2 mmol/l every 5 mins for up to 7 days. After placing the sensor, the iPro2 CGMS needs only a few seconds to initialise. The sensors were placed in the neck area of each cat and covered with a light bandage, as previously described.^12,29^ Calibration was performed every 8–12 h using paired glucose measurements obtained with a validated portable blood glucose meter (AlphaTRAK2; Abbott Animal Health).^30–32^ Caregivers and owners were also instructed to record the timing of insulin treatment, feeding and activity. Any problems during sensor placement, any adverse effects of the iPro2 or any malfunctioning of the device were documented.

The recorded glucose data were uploaded and analysed using CareLink iPro Software (Medtronic). Daytime and night-time periods were defined as the 12-h intervals between two insulin injections and/or feeding times. Glucose was measured every 5 mins; thus, the complete data included 144 diurnal and 144 nocturnal glucose measurements.

### Statistical analysis

Twenty-four-hour glucose curves were included if all 144 measurements recorded during the day and all 144 measurements recorded at night were available; those with missing data were excluded. For each healthy and diabetic cat, mean, minimum and maximum glucose concentrations, SD and CV were calculated for both daytime and night-time periods; data were reported as median and range. In the case of multiple curves being available, the mean of the means was calculated for all parameters except for the minimum and maximum. Hypoglycaemia (nocturnal and diurnal) was defined as a glucose concentration below 3.5 mmol/l, and the percentage of hypoglycaemic episodes was calculated for each cat as the proportion of glucose measurements below this level relative to the total number of readings. In addition, diabetic cats were categorised into two groups based on their fructosamine concentrations. Well-controlled diabetic cats were defined as those without clinical signs of DM (stable weight, normal appetite and drinking behaviour) and fructosamine concentration below 450 µmol/l, while those with persisting clinical signs and fructosamine concentrations above 450 µmol/l were classified as moderately to poorly controlled. For each variable, comparisons between days and nights were performed within healthy and within diabetic cats using the Wilcoxon paired signed-rank test. Comparisons between healthy and diabetic cats, as well as between well-controlled and moderately to poorly controlled diabetic cats, were performed using the Mann–Whitney U-test. Significance was defined as *P* <0.05. GraphPad Prism version 7.04 (GraphPad Software) was used for analysis.

## Results

### Animals

The six healthy cats were castrated males, with a median age of 3.7 years (range 3.4–3.7) and a median body weight of 5.0 kg (range 4.7–5.9). Throughout the study period, they were housed in small groups within a large enclosure, allowing free movement, and they were fed a commercial dry food twice daily to maintain stable body weight.

A total of 16 diabetic cats with at least one paired curve (ie, one day and one night) were initially considered. Five were excluded because glucose measurements were missing for either the day or night. One cat was excluded because the paired curves were recorded during remission. In total, 10 cats were eventually included in the study; among them, four were castrated males and six spayed females. Eight were domestic shorthairs and two were Burmese cats. Their median age was 11.0 years (range 7.0–15.0) and the median body weight was 4.7 kg (range 3.3–8.4). These cats were kept in their home environment throughout the study and received diabetic-specific commercial food twice daily (Diabetes Management; Purina, or Diabetic; Royal Canin). The median duration of DM treatment among the cats was 18 months (range 4 months to 3 years).

The insulin treatment included glargine (Lantus; Sanofi) in seven cats, protamine zinc insulin (ProZinc; Boehringer Ingelheim) in two cats and degludec (Tresiba; Novo Nordisk) in one cat; the seven cats treated with glargine received a median insulin dose of 0.3 U/kg q12h (range 0.1–0.6), the two cats treated with protamine zinc received 0.1 U/kg and 0.4 U/kg q12h, respectively, and the cat treated with degludec received 0.2 U/kg q12h. The insulin dosages in each cat were not changed during the study. Based on clinical signs and the fructosamine concentration cutoff of 450 µmol/l, five cats were classified as well-controlled and five cats were classified as moderately to poorly controlled.

### Continuous glucose measurement

Placement of the CGMS iPro2 was easily performed in all cats. The only adverse effects were mild skin reactions (redness) observed immediately after removal of the sensor from the neck. These reactions healed within a few days without requiring special treatment. However, all but three cats had already pulled out the sensor after 3 days; the median duration the sensors remained in place was 2 days (range 1–5).

### CGMS data

Glucose data from 3 days and 3 nights were included for each healthy cat. The amount of data from every cat varied for the diabetic cats. Four cats had 1 day and 1 night, two cats had 2 days and 2 nights, one cat had 3 days and 3 nights, two cats had 4 days and 4 nights, and one cat had 5 days and 5 nights.

In the six healthy cats, mean, minimum and maximum glucose concentrations showed no significant diurnal-nocturnal differences. However, SD and CV were significantly higher at night (Table 1). Of the healthy cats, five (83.3%) had low diurnal as well as low nocturnal values (<3.5 mmol/l); among these, two had nocturnal glucose levels below the detection limit (2.2 mmol/l): one cat in 2/3 nights and the other cat in 1/3 nights. Overall, the frequency of these episodes was not different between daytime and night-time (*P* = 0.33). See Table 1 for median and range glucose values in healthy cats during daytime and night-time periods.

**Table 1:** Comparison of glucose variables between day and night in healthy cats

Glucose	Day	Night	*P*
Mean (mmol/l)	4.3 (3.5–5)	4.5 (2.8–5.4)	0.522
Minimum (mmol/l)	3.3 (2.3–4.3)	3.3 (2.2–4.1)	0.927
Maximum (mmol/l)	5.2 (4.1–6.3)	5.7 (4.1–8.8)	0.129
SD (mmol/l)	0.4 (0.2–0.6)	0.6 (0.2–0.8)	0.037
CV (%)	10 (4–16)	13 (5–23)	0.041

Data are median (range)

CV = coefficient of variation

In the 10 diabetic cats, mean, minimum and maximum glucose concentrations, as well as SD and CV, showed no significant diurnal-nocturnal differences (Table 2).

**Table 2:** Comparison of glucose variables between day and night in diabetic cats

Glucose	Day	Night	*P*
Mean (mmol/l)	10 (3.5–19.7)	11.3 (4.3–19.4)	0.639
Minimum (mmol/l)	5.4 (3.4–15.4)	7.0 (4.1–17.1)	0.105
Maximum (mmol/l)	14.8 (4.7–22.2)	15 (4.9–22.2)	0.965
SD (mmol/l)	2.1 (0.3–4.9)	1.9 (0.2–4.9)	0.729
CV (%)	19 (5–43)	16 (4–45)	0.374

Data are median (range)

CV = coefficient of variation

The comparison between healthy and diabetic cats showed that mean, minimum and maximum glucose concentrations, and SD were significantly higher in the diabetic group, during both the daytime and night-time. However, CV was significantly higher in diabetic than in healthy cats during the daytime but did not differ at night (Tables 3 and 4).

**Table 3:** Comparison of glucose variables between diabetic and healthy cats during the day

Glucose	Healthy cats	Diabetic cats	*P*
Mean (mmol/l)	4.3 (3.5–5)	10 (3.5–19.7)	<0.001
Minimum (mmol/l)	3.3 (2.3–4.3)	6.5 (2.6–16.7)	<0.001
Maximum (mmol/l)	5.2 (4.1–6.3)	14.8 (4.7–22.2)	<0.001
SD (mmol/l)	0.4 (0.2–0.6)	2.1 (0.3–4.9)	<0.001
CV (%)	10 (4–16)	19 (5–43)	0.005

Data are median (range)

CV = coefficient of variation

**Table 4:** Comparison of glucose variables between diabetic and healthy cats at night

Glucose	Healthy cats	Diabetic cats	*P*
Mean (mmol/l)	4.5 (2.8–5.4)	11.34 (4.3–19.4)	<0.001
Minimum (mmol/l)	3.3 (2.2–4.1)	8.1 (2.2–17.1)	<0.001
Maximum (mmol/l)	5.7 (4.1–8.8)	15 (4.9–22.2)	<0.001
SD (mmol/l)	0.6 (0.2–0.8)	1.9 (0.2–4.9)	<0.001
CV (%)	13 (5–23)	16 (4–45)	0.682

Data are median (range)

CV = coefficient of variation

Among diabetic cats, five had moderately to poorly controlled DM and five were well-controlled. During the daytime, the moderately to poorly controlled diabetic cats exhibited significantly higher mean and maximum glucose concentrations, as well as significantly higher SD, while minimum glucose concentrations and CV did not differ (Table 5). At night, the former had significantly higher mean, minimum and maximum glucose concentrations, while SD and CV remained the same (Table 6).

**Table 5:** Comparison of glucose variables based on fructosamine concentrations in diabetic cats during the day

Glucose	<450 µmol/l	>450 µmol/l	*P*
Mean (mmol/l)	5.8 (4.3–14.1)	14.9 (10–17.8)	0.046
Minimum (mmol/l)	4.9 (3.4–9.8)	6.1 (3.9–15.4)	0.691
Maximum (mmol/l)	7.4 (5.1–19.9)	20 (19.5–22.2)	0.032
SD (mmol/l)	0.6 (0.4–2.7)	3.7 (0.9–3.8)	0.032
CV (%)	10.5 (7.4–21.5)	25.5 (5.1–37.2)	0.135

Data are median (range)

CV = coefficient of variation

**Table 6:** Comparison of glucose variables based on fructosamine concentrations in diabetic cats at night

Glucose	<450 µmol/l	>450 µmol/l	*P*
Mean (mmol/l)	6.3 (4.8–16.2)	18.3 (11.6–19.4)	0.032
Minimum (mmol/l)	4.5 (4.1–10.5)	13.4 (7.7–17.1)	0.032
Maximum (mmol/l)	7.7 (5.4–21.5)	22.2 (17.7–22.2)	0.032
SD (mmol/l)	0.6 (0.3–4.2)	2.4 (0.9–4.2)	0.460
CV (%)	10.9 (6.9–45.2)	12.6 (4.9–26.5)	1.000

Data are median (range)

CV = coefficient of variation

Hypoglycaemic episodes with glucose values below 3.5 mmol/l were observed in four diabetic cats; no clinical sign related to hypoglycaemia was reported. Two cats experienced diurnal hypoglycaemic episodes only. Of these, one cat had 4.2% of its total glucose measurements classified as hypoglycaemic, while the other cat had 2.5%. The lowest values observed were 3.1 mmol/l and 2.9 mmol/l, respectively. One cat had only nocturnal episodes with one single nocturnal hypoglycaemic episode lasting 105 mins out of 4 nights, accounting for 2.4% of all recorded glucose values. The lowest value for this cat fell below the detection limit (<2.2 mmol/l). One cat had both diurnal and nocturnal hypoglycaemic episodes. During the diurnal period, 16.7% of its glucose measurements on one day and 42.3% on another day were classified as hypoglycaemic, with the lowest values recorded as 2.8 mmol/l and 2.6 mmol/l, respectively. During the nocturnal period, hypoglycaemic values accounted for 3.5% of the total night-time measurements, with the lowest observed glucose value being 3.4 mmol/l. Overall, three (30%) diabetic cats had hypoglycaemic episodes during the daytime and two (20%) during the night; hence, their frequency did not differ (*P* = 0.456).

Five diabetic cats showed glucose values above 22.2 mmol/l, with 1%, 4%, 7%, 8% and 11% of the measurements above the upper threshold during both days and nights. Taking into account all 10 diabetic cats included in the analysis, 3.1% of glucose measurements were greater than 22.2 mmol/l.

## Discussion

The results of the present study corroborate the previous observation: that the CGMS used is easily applicable to cats, well-tolerated and useful to non-invasively obtain glucose values over several days. Using this approach, nocturnal measurements were possible, and the obtained values were not affected by the stress and pain of venipuncture or earlobe puncture. However, not all measurements were consistently transmitted, and although the device was intended to measure over 7 days, this duration was not reached in most cats. Premature termination of monitoring was mainly due to the cats removing the device themselves. In addition, because of the retrospective nature of the monitoring system, any decision to modify insulin treatment could only have been made after its removal. Nevertheless, the device proved useful for assessing continuous glucose values over several days and, in particular, for studying glucose values at night, which was the primary goal of this study.

Three out of five hypoglycaemic episodes in the 10 diabetic cats were observed in the daytime and two occurred at night, with the lowest nocturnal value in one cat being below the detection limit (<2.2 mmol/l). This finding highlights that although daytime glucose curves may detect many hypoglycaemic events, the use of a CGMS over a 24-h period can be crucial to identify intermittent nocturnal hypoglycaemia.

Interestingly, healthy cats exhibited significantly higher nocturnal glycaemic variability based on SD and CV, whereas diabetic cats showed no diurnal-nocturnal differences in glucose concentrations or variability. This contrasts with humans, where nocturnal hypoglycaemia is more prevalent, suggesting species-specific differences in circadian glucose regulation. Cats, being crepuscular, may naturally exhibit different metabolic patterns compared with diurnal humans. It cannot be excluded that the influence of circadian rhythm-associated hormones on blood glucose concentration is impaired in diabetic cats. In healthy cats, alpha-melanocyte-stimulating hormone (alpha-MSH) is increased after the onset of darkness, and since it induces peripheral insulin resistance in rodents, it may contribute to the higher glycaemic variability observed in our purpose-bred cats at night. Therefore, if diabetic cats have lower alpha-MSH levels, day–night differences would be less apparent.^33,34^

Not unexpectedly, diabetic cats with moderately to poorly controlled disease showed higher values for most glucose-related parameters compared with well-controlled cases, both during the day and at night. Among the measures of glycaemic variability included in this study, diurnal SD was higher in the former group, likely due to less predictable glucose levels associated with inadequate DM management. However, the absence of a similar finding at night remains unexplained.

It is worth noting that the minimum glucose concentration in healthy cats was lower than the common reference interval. It is possible that healthy cats, in their home environment and without the stress associated with blood sampling, have lower glucose values than what is generally considered normal. However, a false measurement obtained by the device should be considered when evaluating hypoglycaemia in cats using a CGMS.

Despite the drawbacks in sensor retention, the CGMS proved valuable in assessing glucose fluctuations, particularly at night. Newer CGMS devices, such as the Freestyle Libre (Abbott), enable rapid adjustments to insulin therapy, reducing the risk of both hypoglycaemia and hyperglycaemia. This approach can lead to faster and more effective glycaemic control in diabetic cats compared with frequent blood sampling, while also improving owner compliance. Integrating such technologies into routine veterinary practice is expected to considerably improve the management of DM in feline patients.

### Study limitations

The study limitations include the small number of cats, the different duration of DM, and the different insulin types and diets among diabetic cats; in particular, differences in insulin pharmacokinetics and durations of action between individuals might have influenced glucose variability and hypoglycaemic episodes. In addition, two Burmese cats, a breed predisposed to DM, were included, possibly limiting the general applicability of these results. The CGMS device used in this study has detection limits that might have partially influenced the analysis. However, the proportion of glucose measurements outside these detection limits was minimal and unlikely to have a relevant impact on the overall results. In light of the aforementioned limitations, this should be considered a pilot study.

## Conclusions

Continuous glucose monitoring revealed increased nocturnal glycaemic variability in healthy cats but not in diabetic cats. In addition, cats with moderately to poorly controlled DM had higher diurnal glycaemic variability than those that were well controlled. Low glucose concentrations occurred in both groups and at any time, emphasising the benefit of monitoring their concentrations throughout the 24-h period. Glucose nadirs in healthy cats may be lower than previously reported. Glycaemic variability may be an additional tool to assess glycaemic control in diabetic cats.
